# Effect of Using Hybrid Polypropylene and Glass Fibre on the Mechanical Properties and Permeability of Concrete

**DOI:** 10.3390/ma12223786

**Published:** 2019-11-18

**Authors:** Abubaker A. M. Ahmed, Yanmin Jia

**Affiliations:** College of Civil Engineering, Northeast Forestry University (NEFU), No.26 Hexing Road Xiangfang District, Harbin 150040, China; abubakerahmed@hotmail.com

**Keywords:** polypropylene fibre, glass fibre, hybrid fibres, mechanical properties, permeability

## Abstract

A comprehensive program of experiments consisting of compression, uniaxial compression, direct shear, flexural as well as splitting tensile and air permeability tests were performed to analyse the effect of the level of fibre dosage and the water–cement ratio on the physical properties of hybrid fibre-reinforced concrete (HFRC). Two types of fibres were studied in terms of their effect on the properties of HFRC. The results indicated that the mechanical properties of concrete were significantly improved by increasing the fibre content. However, increasing the percentage fibre content past a certain peak performance limit (0.9% glass fibre (GF) and 0.45% polypropylene fibre (PPF)) led to a decrease in strength compared to reference mixes. Additionally, the incorporation of hybrid fibres yielded an increase in air permeability in the tested specimens. The results showed that the strength-related properties of HFRC were superior to the properties of single fibre-reinforced concrete.

## 1. Introduction

Unreinforced concrete is brittle in nature, and is characterized by low tensile strength but high compressive strength. Because of this property and a lack of bonding in the concrete matrix at the transition zone, the brittleness increases along with increasing concrete strength. [1], Additionally, concrete is susceptible to early age cracking caused by shrinkage due to the use of mineral additives such as silica fume [2]. The addition of small, short, randomly spaced fibres in the concrete improves its properties and can mitigate its poor resistance to crack growth and brittleness. Combining fibres with concrete can produce a range of materials which possess enhanced tensile strength, elasticity, toughness, and durability. This is accomplished by limiting or controlling the start, spread, or spread persistence of cracks [3,4,5,6,7]. Several types of fibre are available—the predominant types are natural, synthetic, steel, carbon and glass [8,9,10,11,12,13].

Banthia et al. [3] investigated the effect of using macro-steel fibres and micro-cellulose fibres on the flexure and direct shear strength of single and hybrid concrete mixes. Test results for the hybrid composites examined under flexure showed that the cellulose fibre made an active contribution to toughness. When examined under direct shear, the synergy was not uniformly positive across all fibre types and combinations. Gabriel et al. [14] developed a self-consolidating hybrid fibre-reinforced concrete (HFRC) composite with superior flowability and workability incorporating polyvinyl acetate (PVA) fibres and steel fibres. The composite possessed crack control and crack stabilization characteristics by virtue of its toughening mechanisms, and had higher residual strength capacity compared with non-fibrous concrete. Banthia and R. Gupta [15] investigated the effect of steel, polypropylene fibre, and carbon fibre hybridization on high-strength concrete. They found that the addition of micro-fibres enhanced the modulus of rupture (MOR). Additionally, deformed steel micro-fibres were shown to yield concrete of superior toughness than crimped polypropylene microfibers. Maximum synergy was found in the hybridization of crimped polypropylene macro-fibre with micro-fibres of carbon and polypropylene. However, the mix containing self-fibrillating polypropylene micro-fibre showed no synergy at all. Dawood et al. [16] reported that the use of hybrid fibre combinations in reinforced concrete, such as steel fibres and palm fibres, would enhance its flexural toughness and rigidity along with its overall performance.

The mechanical properties of fibre-reinforced concrete are affected by many factors, but the main two factors are the bonding strength between the fibres and the concrete mixture, and the distribution of fibres on the mixture. The distribution of fibres in the mix can be enhanced significantly by adding silica fume, fly ash and slag [17,18].

Studies have shown that permeability in concrete is influenced more by cracks in the mortar binder than by the state of the mortar binder–aggregate interface. Permeability is affected by the width of the cracks in the mortar, and thus bridging the micro cracks before they integrate is a useful method of restricting fluid transport [19]. The use of fibre to reinforce concrete produces impermeable concrete, and this decreases the flow of liquids and gas efficiently even while the concrete is under stress [19,20]. Fibre-reinforced concrete (FRC) has a higher deformation capacity than plain concrete (PC), but the fibres minimize the permeability of the concrete [21]. The presence of fibres in the concrete leads to changes in the concrete’s crack profile. Instead of the formation of a few large cracks, a lot of closely spaced micro cracks form. However, an increase in the fibre volume fraction can also lead to an increase in permeability [21,22]. The level of permeability is dependent on the fibre type and size. Micro-fibres can efficiently decrease concrete permeability, while concrete containing macro-fibres demonstrates an insignificant improvement in concrete permeability [23].

Air permeability tests are frequently used due to their quick results and the fact that the concrete pore structure is unaffected during the test [24,25,26].

A diverse range of field techniques are available for the estimation of concrete air permeability, some of which have become standardized [27,28,29,30].

According to the latest research results, scientists have clearly confirmed the effectiveness of fibres in enhancing the performance of concrete. However, there is still a need for further study of the gas permeability in concrete reinforced with multiple types of fibres. With the increasing prevalence of the use of FRC applications in civil engineering, an understanding of the effects of fibres on the permeability of concrete is becoming increasingly important in order to predict the service life of FRC structures. The aim of this study was to create an improved model of the relationship between fibres and permeability. This element is essential for the design of concrete mixes as construction materials to ensure appropriate structural performance in the final products.

In this study, a comprehensive set of experimental data was generated upon using hybrid polypropylene and glass micro fibres in various volume percentages (0%, 0.45%, 0.9% and 1.35%) and different water/binder ratios (0.30 and 0.35) to check the effect of increasing the amount of binder along with fibre volume on the concrete properties. These properties included compressive strength, split tensile strength, flexural strength and shear strength. Additionally, the air permeability of concrete made with different water/cement ratios (0.30 and 0.35) was also compared with conventional concrete after curing for 28 days.

## 2. Materials and Methods

A program of experimentation was designed to study the compressive, shear, tensile and flexural strength of single and hybrid polypropylene glass fibre-reinforced concrete, in addition to studying the air permeability of all samples. The concrete mixes were designed with two water/cement ratios and different volume fractions (0.0%, 0.45%, 0.9% and 1.35%).

### 2.1. Raw Materials

Ordinary Portland cement, fly ash, silica fume and mineral powder were used in accordance with China national standard GB175-2007.

A dry and clean local river sand was used as the fine aggregate, and a coarse aggregate with a maximum particle size of 20 mm was used in the concrete.

The properties of the glass and polypropylene fibres are shown in Table 1.

### 2.2. Test Methods

To determine the strength performance, for each mixture, fifteen specimens (six 100 × 100 × 100 mm cubes, three 100 × 100 × 400 mm beams, six 100 × 100 × 300 mm beams, and three 150 × 300 mm cylinders) were prepared. These were standard test specimens prepared in accordance with the Chinese standard CECS13:2009 [31]. Three specimens were prepared for each test, and the mean value was used.

The compressive strength and uniaxial compressive strength, shear strength, flexural strength, and splitting tensile strength were tested after curing the concrete for 28 days.

The samples were cured in a curing chamber at 100% relative humidity and a temperature of 20 ± 2 °C.

The compressive test was conducted based on the Chinese standard GB/T50081-2016 [32]. Cubes of side 100 mm and prisms of dimensions 100 mm × 100 mm × 300 mm were produced to measure the compressive strength of concrete mixtures. A hydraulic universal testing machine (WE-30) was used to conduct the compression test with a loading control of 0.5–0.8 MPa/s.

**Figure 1:**
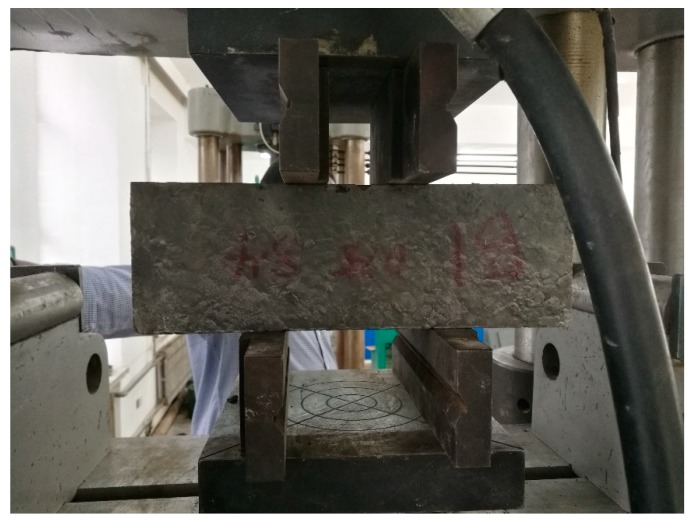
Direct shear test experimental set-up.

The Chinese standard JSCE-G 553-1999 [33] describes a method for generating a pure shear condition which can be used to test samples in the lab, as shown in Figure 1. A prism of dimensions 100 mm × 100 mm × 400 mm was used to estimate shear strength.

The bending test was conducted on prisms of dimensions 100 mm × 100 mm × 400 mm to estimate the flexural strength of each mixture according to GB/T50081-2016. The hydraulic universal testing machine (WE-30) was used to conduct the bending, and the flexural test was conducted with a loading control of 0.5–0.8 MPa/s.

Splitting tensile strength tests were performed on the cylindrical specimens of dimensions 150 × 300 mm following the method in ASTM C496 [34] using a loading rate of 0.8 MPa/min until the samples failed.

Air permeability was measured using a standardized air permeability measurement system (Autoclam, amphora NDT, Belfast, Northern Ireland, UK). To calculate the air permeability index (API), natural logarithms of air pressure were plotted with respect to time. The slope of the last 10 results was designated as the API [35].

The average permeability coefficients were calculated to obtain a representative value of permeability for each specimen.

A JSM-7500F scanning electron microscope (JEOL (BEIJING) CO. LTD., Beijing, China) was used to obtain scanning electron microscopy (SEM) images. At the microstructural level, the bonding surface between the fibres and cement paste can be observed in single or hybrid concrete. The capillary pores and microcracks of concrete can be better understood through the SEM images.

### 2.3. Mixture Proportions

Twenty mixes were designed with water-to-binder ratios of 0.35 and 0.30 and containing a total cementitious material of 370 kg/m^3^ and 430 kg/m^3^, respectively. The control mixture included only cementitious materials (Ordinary Portland cement, fly ash, silica fume and mineral powder) and fine and coarse aggregate, while fibres were added to the remaining mixtures as shown in Table 2.

The percentages of added fly ash, silica fume and mineral powder were 20%, 5% and 10%, respectively, by weight of cement. The concrete mixture proportions are summarized in Table 3.

## 3. Results

The results for the compressive strength, shear strength, flexural strengths, and splitting tensile strength for the cubes and prisms incorporating all mixtures are presented in Figure 2, Figure 3, Figure 4, Figure 5, Figure 6, Figure 7, Figure 8, Figure 9, Figure 10 and Figure 11.

### 3.1. Compressive Strength

**Figure 2:**
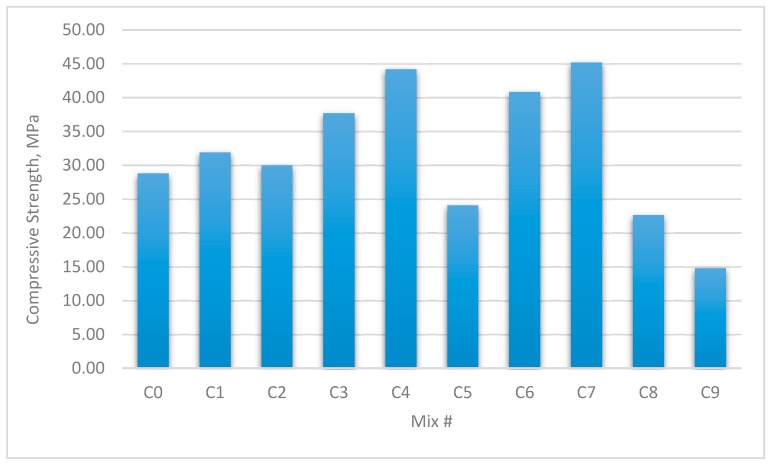
Compressive strengths of concrete cubes with W/B ratio 0.3.

**Figure 3:**
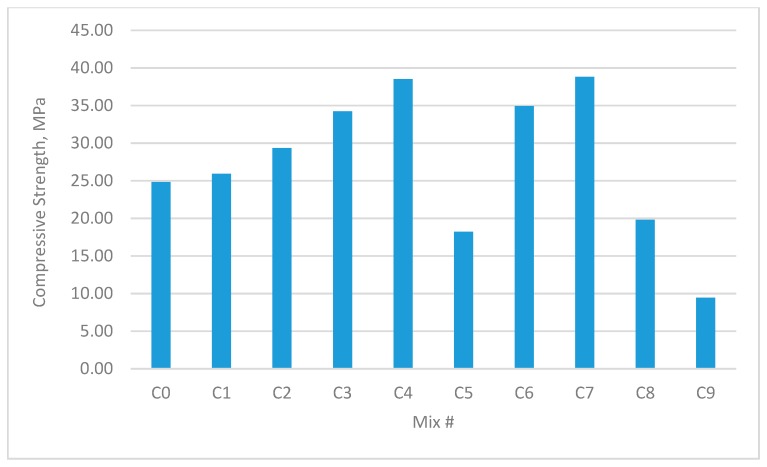
Uniaxial compressive strength of concrete beams with W/B ratio 0.3.

The largest improvement in compression strength for the cubes and prisms was 54.1% and 56.34%, respectively, compared with the reference mix. This occurred in the set C7, which contained 1.35% glass fibre (GF) and 0.0% polypropylene fibre (PPF) with a water-to-binder ratio (W/B) of 0.30. This is shown in Figure 2 and Figure 3.

Upon examining the compressive strength results, it was noted that the strength of the concrete containing GF improved, compared with mixes containing PPF and with the reference mix. This result indicates that the addition of PPF had a small impact on the concrete’s compressive strength. [36].

**Figure 4:**
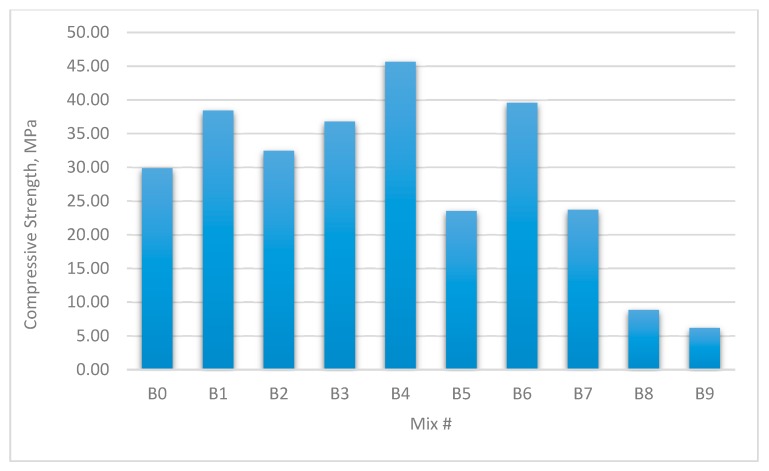
Compressive strengths of concrete cubes with W/B ratio 0.35.

**Figure 5:**
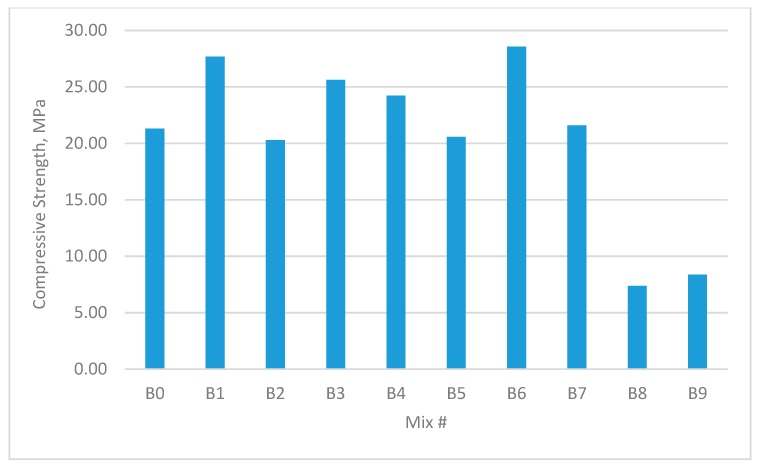
Uniaxial compressive strength of concrete beams with W/B ratio 0.35.

Figure 4 and Figure 5 show the performance of mixes with W/B = 0.35. The maximum increase in cube compressive strength was 52.77%, which occurred in group B4 containing 0.9% GF and 0.0% PPF. The maximum increase in prism uniaxial compressive strength was 34.14%, which occurred in group B6 containing 0.9% GF and 0.9% PPF.

Comparisons between the control mix and the hybrid fibre mix indicated that the use of a low volume fraction of PPF (0.45%) enhanced the compressive strength of the concrete. This was likely because hybrid fibres with different sizes and types offer different restraint conditions [37,38,39]. On the other hand, the compressive strength results showed that the use of more than 0.45% PPF in single fibre mixes reduced the compressive strength. This was due to the low stiffness of the PPF and the displacement of the mortar matrix of the mixes, leading to a reduction in compressive strength.

### 3.2. Shear Strength

**Figure 6:**
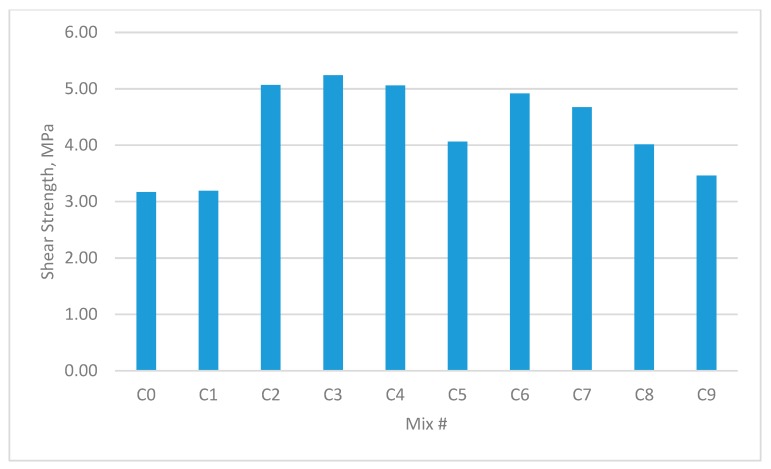
Shear strength of concrete beams with W/B ratio 0.3.

**Figure 7:**
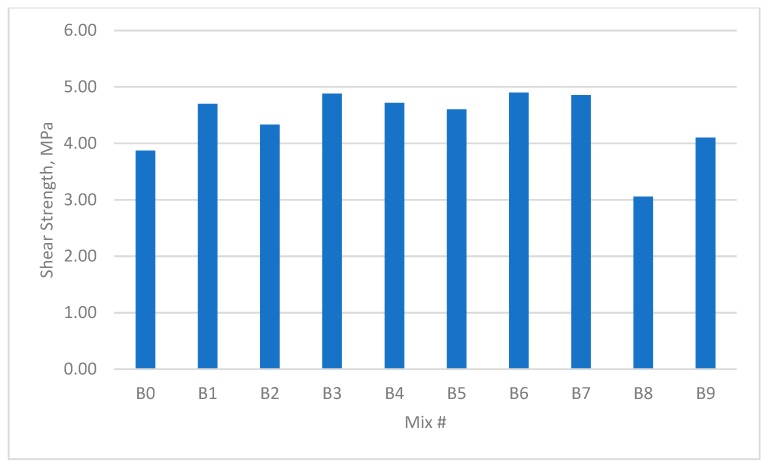
Shear strength of concrete beams with W/B ratio 0.35.

The shear strengths of all samples containing fibres were noted as being higher than the reference mix. This is shown in Figure 6 and Figure 7. Unlike the small improvement seen in compressive strength, the maximum increase in direct shear strength was approximately 65%. The experimental results showed that group C3 as shown in Figure 6, which contained 0.45% GF and 0.45% PPF with a W/B of 0.30, possessed the maximum shear strength amongst all test samples. The increase in GF content resulted in considerable improvements in the shear strength. When the W/B was 0.35, the test results indicated the maximum increase in direct shear strength (approximately 34%) in the B3 group, which contained 0.45% GF and 0.45% PPF.

An increase in the added volume fraction of single and hybrid fibres resulted in this significant enhancement of the direct shear strength. However, the shear strength may decrease due to the volumetric expansion of PPF when it meets water. If the unit weight of PPF exceeds a reasonable range, the extra PPF may cause a nonuniform distribution of the fibres, and thus lead to a decrease in the direct shear strength.

### 3.3. Splitting Tensile Strength

**Figure 8:**
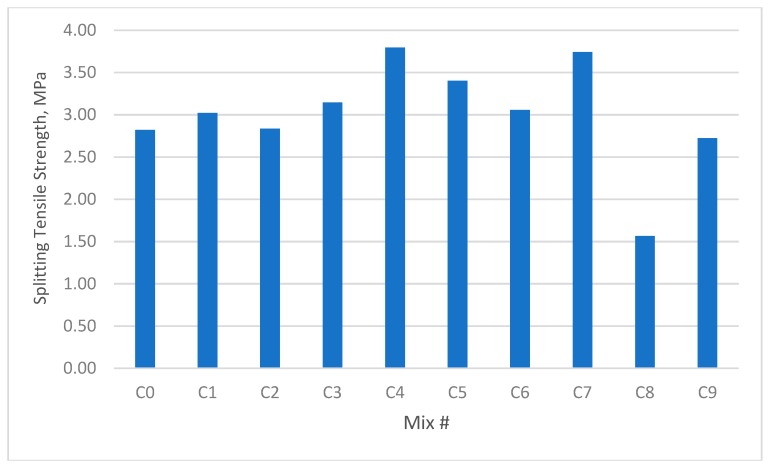
Splitting tensile strength of concrete beams with W/B ratio 0.3.

**Figure 9:**
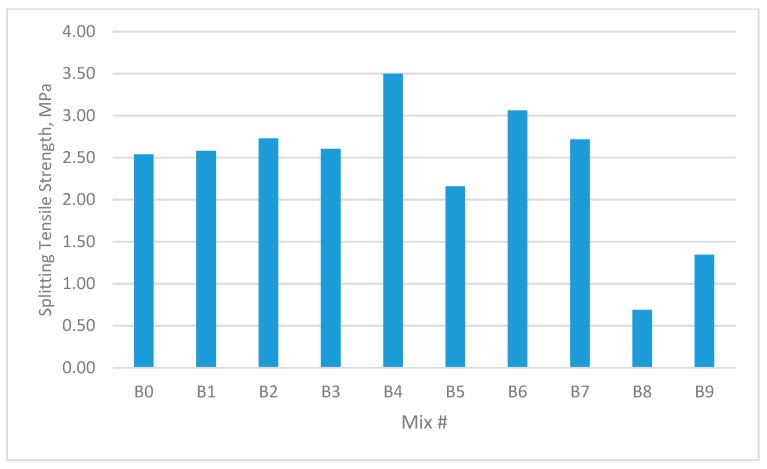
Splitting tensile strength of concrete beams with W/B ratio 0.35.

As can be seen from Figure 8 and Figure 9, the fibre concrete mixes demonstrated enhanced splitting tensile strength in the hybrid form containing GF and PPF, whilst demonstrating a splitting tensile strength reduction of over 45% when they contained only PPF.

The results were similar to the compressive strength tests discussed in the previous section, in that the GF additive yielded the highest splitting tensile strength, which was 44.73% higher than that of the unreinforced control concrete. This occurred in group B4 containing 0.9% GF and 0.0% PPF, while the PPF additive yielded the lowest splitting tensile strength of (−72.8%) compared with reference mix. This occurred in group B8 containing 0.0% GF and 1.35% PPF.

In the second group of mixes with W/B of 0.30, the maximum increase in direct shear strength was approximately 34.65% for group C4 compared with the reference mix. The PPF additive yielded the lowest splitting tensile strength of 44% compared with the reference mix. This occurred in group C8 containing 0.0% GF and 1.35% PPF.

It was clear that the GF additive increased the splitting tensile strength. This was very clear in group #9 (C9 and B9), which contained a larger volume of fibre (1.35% GF and 1.35% PPF). However, the splitting tensile strength of group #9 was higher than that of group #8 (C8 and B8), which had a smaller volume of fibre but did not contain GF (0% GF and 1.35% PPF).

The impact of the added fibres on tensile strength indicated that the interfacial bonding between the fibres and matrix had improved.

### 3.4. Modulus of Rupture (MOR)

Mixes containing single or double fibres showed improvements in flexural strength compared to the equivalent reference plain concrete mix.

**Figure 10:**
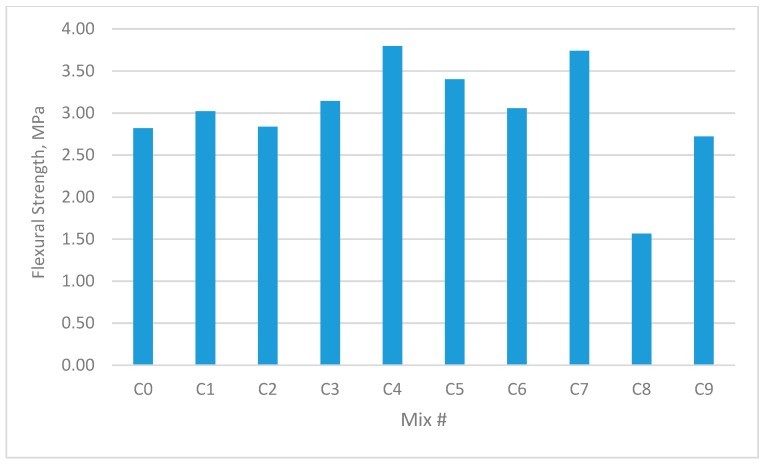
Flexural strength of concrete beams with W/B ratio 0.3.

**Figure 11:**
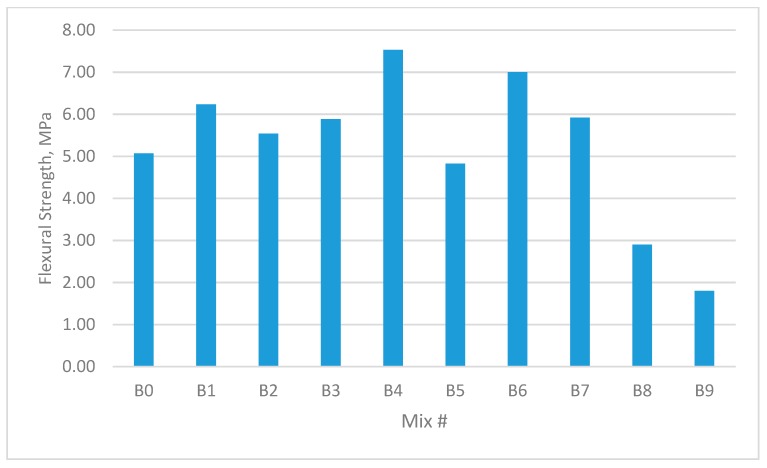
Flexural strength of concrete beams with W/B ratio 0.35.

As shown in Figure 10 and Figure 11, GF concrete showed a 40.0% and 48% increase in flexural strength over the reference mix for samples C4 and B4, respectively, while the PPF concrete showed a decrease of −65% in both C9 and B9, respectively, which contained 1.35% GF and 1.35% PPF. The increase in the MOR resulted principally from the fibres which acted as lines or wires that resisted the micro-cracks in the tension zones of the samples.

As noted in the discussion of the compressive and splitting tensile strength tests, the GF and PPF additives improved the MOR for the hybrid fibre concrete compared to the control mixes. This could be due to improvements in the toughness matrix, compactness and homogeneity of fibre distribution in hybrid fibre concrete.

### 3.5. Mechanical Performance Percentage

To calculate the mechanical performance of concrete mixes, a certain weight was assumed for each strength parameter according to their importance in the design of concrete.

As compressive strength is an important parameter to determine the performance of the material during service conditions, added to the fact that concrete is known for its compressive strength, the highest weight (4 of 4) was considered for compressive strength, followed by flexural strength (2 of 4), and split tensile strength (1 of 4).

The mechanical performance percentage of plain and fibre concrete mixes was calculated using the equation proposed by Ali et al. [40].

The mechanical performance (MP) of each mixture was evaluated using Equation (1):

(1) $$\mathrm{MP}\;\left(\%\right)=\frac{4\times \frac{{\;f}_{\mathrm{compressive}-\mathrm{mix}}}{f_{\mathrm{compressive}-\mathrm{CON}}}+2\times \frac{f_{\mathrm{flexural}-\mathrm{mix}}}{f_{\mathrm{flexural}-\mathrm{CON}}}+\frac{f_{\mathrm{split}-\mathrm{mix}}}{f_{\mathrm{split}-\mathrm{CON}}}}{7}\times 100,$$

where: “CON” is control or reference mix, and “mix” is the particular mix whose mechanical performance is being evaluated.

**Figure 12:**
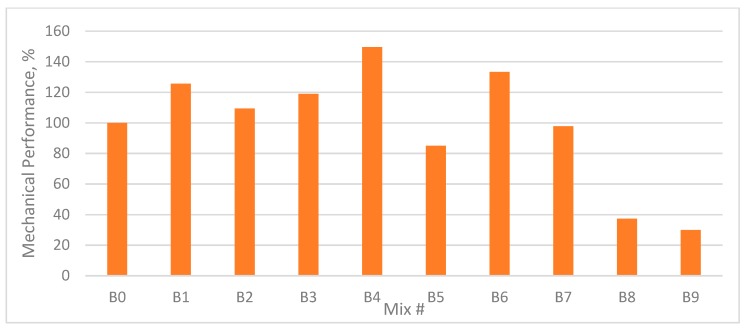
Mechanical performance of each mixture with respect to control mix (B0) with W/B 0.35.

**Figure 13:**
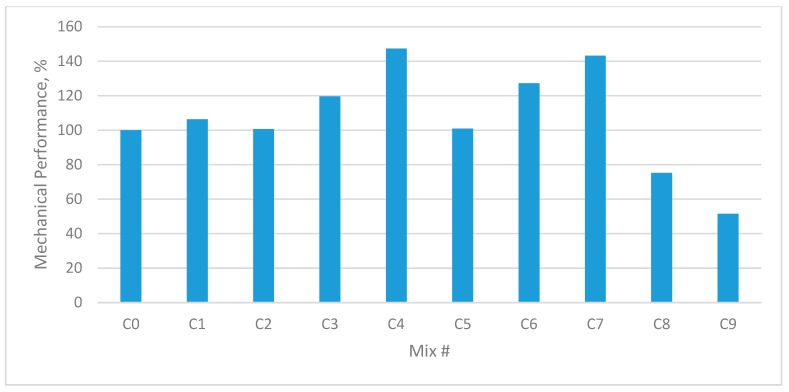
Mechanical performance of each mixture with respect to control mix (C0) with W/B 0.30.

As shown in Figure 12 and Figure 13, the MP (%) for each mix was calculated by incorporating the results of the compressive strength, flexural strength, and tensile strength tests.

It was noted that all mixes containing fibre in single or hybrid form exceeded the reference mix MP until the fibre content reached 0.45%. When the fibre content increased over that volume fraction, the MP started to decrease to the minimum value noted in sample #9 (C9, B9).

The maximum MP was noted in sample #4, which contained 0.9% GF and 0% PPF. This was because mixes B4 and C4 demonstrated high compressive strength, flexural strength, and tensile strength.

### 3.6. Air Permeability of Fibre Concrete

The objective of this test was to examine the impact of hybrid fibre on air permeability volume by examining the permeability of both plain concrete and fibre concrete.

**Figure 14:**
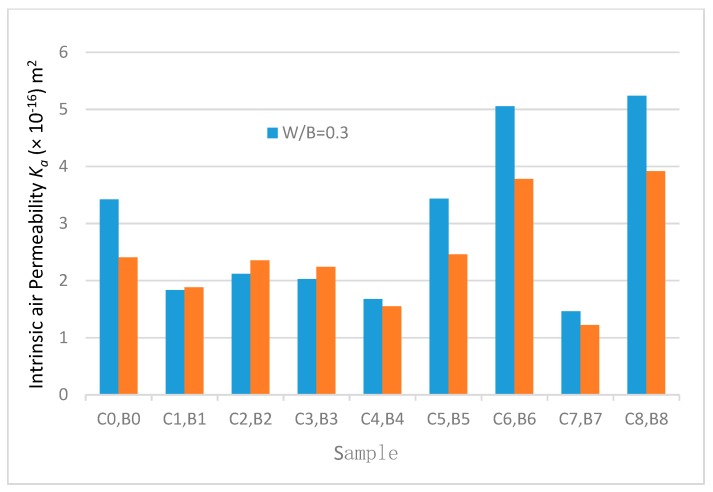
Intrinsic air permeability with W/B 0.35 and W/B 0.30.

**Figure 15:**
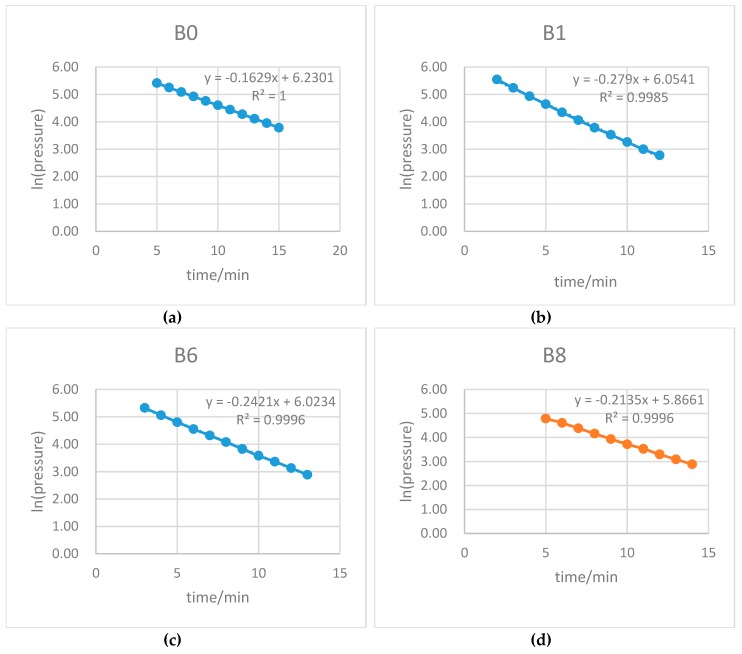
Behaviour of samples during permeability test (W/B = 0.35) for: (**a**) sample B0, (**b**) sample B1, (**c**) sample B6, and (**d**) sample B8.

**Figure 16:**
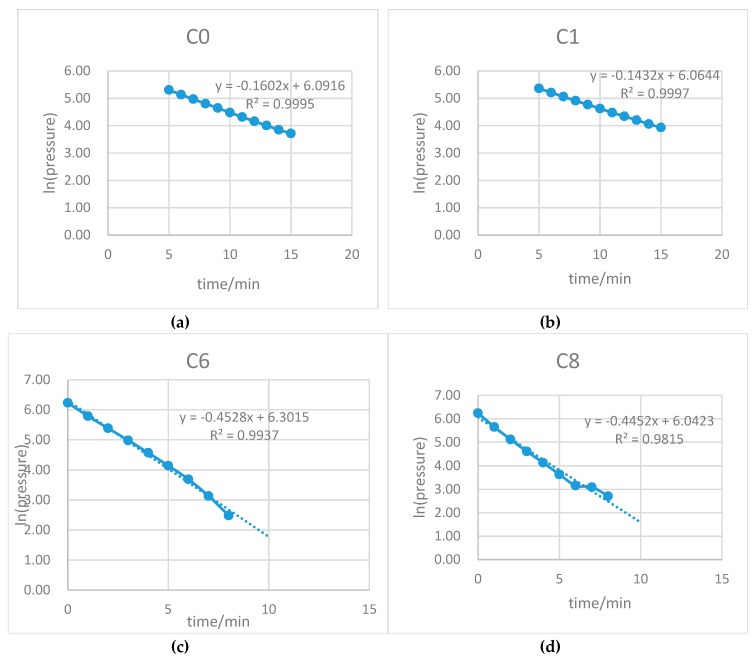
Behaviour of samples during permeability test (W/B = 0.30) for: (**a**) sample C0, (**b**) sample C1, (**c**) sample C6, and (**d**) sample C8.

The permeability test results for the fibre concrete and the plain concrete are shown in Table 4 and Figure 14. The behaviour of samples during the permeability test is shown in Figure 15 and Figure 16. Due to the large variability normally associated with water permeability results, it is often advisable to compare permeability data based on ratios [41].

For mixes of plain concrete (C0 and B0), the results of tests showed that an increase in W/B ratio from 0.30 to 0.35 resulted in an increase in air permeability [42].

W/B ratio and the content and type of supplementary cementitious materials have a significant impact on air permeability. An improvement in the impermeability of concrete containing fly ash is due to pozzolanic reaction effects and changes in the phase composition of hardened cement paste [35].

When samples containing 1.35% GF and 1.35% PPF were used (i.e., when C9 and B9 samples were tested), the tests could not be completed because there was insufficient pressure in the samples. When samples containing 1.35% GF and 0% PPF (i.e., C7 and B7) were used, the permeability coefficient significantly decreased to maximum values of 57% and 50%, respectively. The reduction in the permeability of concrete due to fibre reinforcement is congruent with the findings of Sanjuan et al. [43]. The permeability coefficients of the concrete containing 0.45% and 1.35% PPF were higher than those of the concrete specimens containing 0.45% and 1.35% GF. Therefore, it could be concluded that GF additives can improve the impermeability of concrete. The reduction in permeability associated with fibre reinforcement is due to the fact that the fibres produce mixture stiffening, reduce the settlement of aggregates and decrease bleeding. These lead to a decrease in the ease with which flow can occur through the material. On the other hand, GF and PPF are expected to improve water retention in the cement binder and reduce shrinkage. This is expected to produce a more solid material with less internal cracking. The capability of fibre to reduce the permeability of concrete is affected by many factors, such as the mixture composition, fibre type and dimensions and the nature of the fibre [44].

Table 4 shows the protective quality of the air permeability of concrete.

For structural concrete, the Autoclam air permeability index (API) in ln(pressure)/min can be expressed in terms of intrinsic air permeability, *K_a_*, which is given by using the formula in the AutoCLAM permeability system operating manual:

(2) $$K_{a}\left(m^{2}\right)={\left(\mathrm{API}\right)}^{0.8754}\times 8\;.395\times 10^{-16}$$

The intrinsic air permeability results for the fibre concrete and the plain concrete are shown in Table 4 and Figure 14.

### 3.7. Microstructure Observation

**Figure 17:**
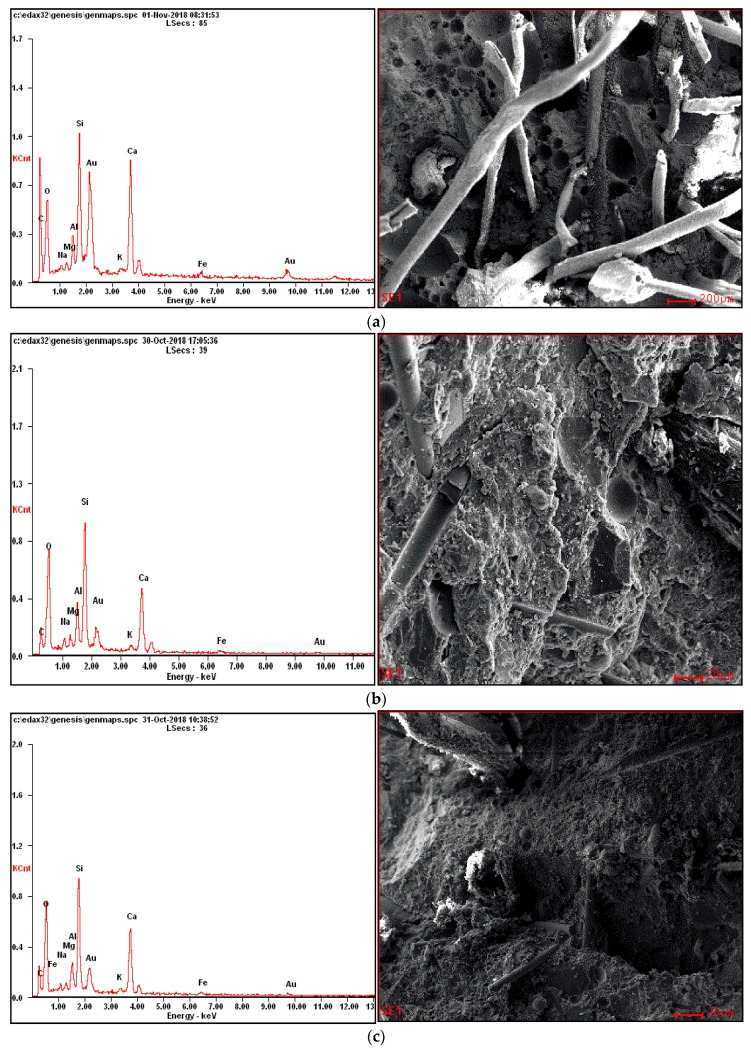

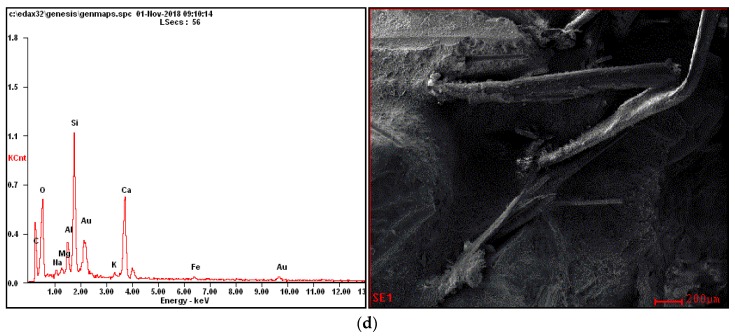
SEM images and energy dispersive X-ray tests of the fibre in the concrete matrix for: (**a**) sample B8, (**b**) sample B3, (**c**) sample C6, and (**d**) sample C9.

The microstructure of concrete is very important in terms of the concrete’s strength and durability. Figure 17 shows scanning electron microscope (SEM) images of selected mixtures using different W/B ratios and fibre dosages after 28 days of concrete curing, and therefore arrest of hydration was not necessary.

The figure shows that the hybrid fibre and single fibre concrete possessed more compact internal structure compared to the control mix. This was due to the fibre having a great affinity for water. By restricting the evaporation of water required for cement hydration from the structure of the mortar samples during the curing period, the cement binder around the fibre could properly hydrate and fill part of the space between the fibre and the binder. This was very conducive to strength development in the concrete.

It could be plainly observed that there was coherence in the fibres and the binder. It could be concluded that the addition of fibres affected the mortar setting, since it could be seen that the mortar was set nearby the fibres without producing further notable porosity. Hence, the fibres were embedded in the set mass and demonstrated good adhesion between themselves and the mortar. This was verified by the mechanical behaviour. On the other hand, the concrete samples containing higher doses of single or hybrid fibre (B8 and B9) demonstrated low compactness, large porosity and some micro-cracks, which all affected the strength development of the concrete.

When the volume of fibre increased, the GF—being the fibres with the highest density—took up a larger volume of the composite material, which led to further deterioration of the mechanical properties. Based on the above analyses, to obtain superior strength and mechanical properties, the application ratios of GF and PPF should be designed so as not to exceed approximately 0.9% and 0.45% of the PC, respectively.

## 4. Conclusions

1. The addition of GF and PPF, as single fibres or in hybridization, of up to 0.45% volume fraction of the fibre could improve the mechanical properties of concrete.
2. The mechanical properties of concrete were significantly increased, and the maximum compressive strength slightly increased by increasing the content of GF up to 1.35% in the tested concrete.
3. A further increase in PPF of higher than 0.45 volume percentage did not increase the ultimate strength, but yielded significantly more ductile bond behaviour.
4. After adding GF and PPF to concrete, it could be seen from the SEM images that the internal pores of the concrete were filled with fibres, the porosity decreased, the structure became more compact and the fibres shared the internal forces experienced by the concrete, thus improving the strength and toughness of concrete and its rheological properties.
5. This investigation demonstrated that hybrid fibre could aid in enhancing the durability of concrete by reducing the permeability at low fibre volume.
6. It can be concluded that GF additives could improve the impermeability of concrete in samples containing up to 1.35% GF. On the other hand, the permeability coefficients of the concrete containing 0.45% and 1.35% PPF were higher than those of the concrete specimens containing 0.45% and 1.35% GF and hybrid fibre (GF + PPF). Therefore, we concluded that GF additives can improve the impermeability of concrete.
7. With the increasing prevalence of the use of FRC applications in civil engineering, an understanding of the effects of fibres on the permeability of concrete is becoming increasingly important in order to predict the service life of FRC structures. This study creates an improved model of the relationship between fibres and permeability. This element is essential for the design of concrete mixes as construction materials to ensure appropriate structural performance in the final products.

## Figures and Tables

**Table 1:** Physical properties of fibres.

Fiber Type	Fibre Length (mm)	Density (g/cm^3^)	Tensile Strength (MPa)	Elastic Modulus (GPa)	Melting Point (°C)
Glass fibre	15	2.36	>450	72	160
Polypropylene	12	0.91	>400	>3.5	160–170

**Table 2:** Volume percentage of fibres in concrete mixes. PP: polypropylene.

Fiber Type	B0, C0	B1, C1	B2, C2	B3, C3	B4, C4	B5, C5	B6, C6	B7, C7	B8, C8	B9, C9
GF %	0	0.45	0	0.45	0.9	0	0.9	1.35	0	1.35
PPF %	0	0	0.45	0.45	0	0.9	0.9	0	1.35	1.35

**Table 3:** Mixture proportion (kg/m^3^). W/B: water-to-binder ratio.

Designation	W/B	Cement	Flay Ash	Silica Fume	Mineral Powder	Water	Sand	Gravel
B	0.30	240	74	18.28	37	129	677.44	1204.41
C	0.35	279.50	86	21.50	43	129	655.26	1165.19

**Table 4:** Protective quality of the air permeability of concrete.

Sample	Autoclam Air Permeability Index: ln(pressure)/min	Quality	Sample	Autoclam Air Permeability Index: ln(pressure)/min	Quality
C0	0.359	good	B0	0.240	good
C1	0.176	good	B1	0.181	good
C2	0.207	good	B2	0.234	good
C3	0.197	good	B3	0.221	good
C4	0.159	good	B4	0.145	good
C5	0.360	good	B5	0.246	good
C6	0.560	poor	B6	0.402	good
C7	0.136	good	B7	0.111	good
C8	0.583	poor	B8	0.418	good
C9	N/A	-	B9	N/A	-
